# Analgesic Effect of *Zanthoxylum nitidum* Extract in Inflammatory Pain Models Through Targeting of ERK and NF-κB Signaling

**DOI:** 10.3389/fphar.2019.00359

**Published:** 2019-04-24

**Authors:** Fenfen Qin, Han Zhang, Anlong Liu, Qisheng Wang, Qinmei Sun, Shengfeng Lu, Qian Li, Hongwei Guo, Xing Liu, Zhigang Lu

**Affiliations:** ^1^College of Pharmacy, Nanjing University of Chinese Medicine, Nanjing, China; ^2^Key Laboratory of Acupuncture and Medicine Research of Ministry of Education, Nanjing University of Chinese Medicine, Nanjing, China; ^3^First Clinical Medical College, Nanjing University of Chinese Medicine, Nanjing, China; ^4^College of Pharmacy, Guangxi Medical University, Nanning, China; ^5^College of Pharmacy, Shanxi University of Chinese Medicine, Taiyuan, China; ^6^Jiangsu Key Laboratory for Pharmacology and Safety Evaluation of Chinese Materia, Nanjing University of Chinese Medicine, Nanjing, China

**Keywords:** *Zanthoxylum nitidum*, Liang Mianzhen, inflammatory pain, pro-inflammatory cytokines, NF-κBp65, p-ERK1/2, peripheral and central mechanism

## Abstract

**Background:**

*Zanthoxylum nitidum (Roxb.) DC.*, also named Liang Mianzhen (LMZ), one kind of Chinese herb characterized with anti-inflammatory and relieving pain potency, which is widely used to treat injuries, rheumatism, arthralgia, stomach pain and so on in China. But its mechanism related to the anti-hyperalgesic has not been reported. The aim of this study was to investigate the analgesic activity of Liang Mianzhen on mice with Complete Freund adjuvant (CFA)-induced chronic inflammatory pain. Meanwhile, the peripheral and central mechanisms of analgesic effect of Liang Mianzhen were further examined via observing the effects of Liang Mianzhen on the signal pathway associated with inflammatory induced hyperalgesia.

**Methods:**

The inflammatory pain model was established by intraplantar injection of CFA in C57BL/6J mice. After 1 day of CFA injection, the mice were treated with LMZ (100 mg/kg) for seven consecutive days, and the behavioral tests were measured after the daily intragastric administration of LMZ. The morphological changes on inflamed paw sections were determined by hematoxylin eosin (HE) staining. Changes in the mRNA expression levels of tumor necrosis factor (TNF-α), interleukin-6 (IL-6), interleukin-1β (IL-1β) and nuclear factor κB p65 (NF-κBp65) were measured on day seven after CFA injection by using real-time quantitative PCR analysis and enzyme linked immunosorbent assay (ELISA) method, respectively. Moreover, immunohistochemistry and western blotting were used to detect extracellular regulated protein kinases 1/2 (ERK1/2) and NF-κB signal pathway activation.

**Results:**

The extract of LMZ (100 mg/kg) showed a significant anti-inflammatory and analgesic effect in the mice model. The paw edema volume was significantly reduced after the administration of LMZ compared to CFA group, as well as the paw tissues inflammatory damage was relived and the numbers of neutrophils in mice was reduced significantly. The CFA-induced mechanical threshold and thermal hyperalgesia value were significant improved with LMZ treatment at day three to day seven. We also found the mRNA levels of TNF-α, IL-1β, IL-6 and NF-κBp65 were down-regulate after 7 days from the LMZ treatment compared to CFA group. Meanwhile, LMZ significantly suppressed over-expression of the phosphorylation of ERK1/2 and NF-κBp65 in peripheral and central.

**Conclusion:**

The present study suggests that the extract of LMZ attenuates CFA-induced inflammatory pain by suppressing the ERK1/2 and NF-κB signaling pathway at both peripheral and central level.

## Introduction

Pain is one of the most common symptoms in clinical practice, and inflammatory pain is the most important type of pain (Patapoutian et al., 2009). Inflammatory pain is a growing global health problem. In generally, there is a common course of progression in inflammation and pain share. When patients experience inflammation, they may develop hyperalgesia or allodynia to various mechanical, thermal, or chemical stimuli (Sommer et al., 2001; Fongang et al., 2017). Inflammatory pain belongs to the category of chronic pain, which is a persistent pain state caused by peripheral tissue damage mediated by a variety of factors (including trauma, bacterial, viral infection and surgery, etc.,) and is one of the most common types of clinical pain (Milligan et al., 2005). Inflammatory pain diseases often occur simultaneously with other diseases (trauma, rheumatoid arthritis, osteoarthritis, etc.) and show complex paralgesia and hyperalgesia. CFA is often used in the induction of various chronic inflammatory pain models. The study of peripheral and central mechanisms of inflammatory pain has been focused by the international research (Xu et al., 2010; Atzeni et al., 2018; Rosas et al., 2018). Numerous studies have shown that many peripheral and central nervous structures and a variety of chemicals are involved in the formation and regulation of inflammatory pain (Vane and Botting, 1995).

Tissue inflammation stimulates the production of cytokines and enzymes that cause inflammatory pain. The chemical mediators (TNF-α, IL-1β, IL-6, and growth factors) released during inflammation can lead to nociceptor sensitization (Planells-Cases et al., 2005). Nociceptor sensitization lowers neuronal thresholds through activation of NF-κB, CREB, MAPKs, and various other transcription factors (Ishii, 2013). Inflammatory mediators such as TNF-α, IL-1β, and IL-6 are key molecular that stimulate peripheral receptors, and their number and activity are regulated by a variety of signal transduction pathways during inflammation, which contributes to development and progression of inflammatory pain (Zhang and Huang, 2006).

Increasing numbers of studies have shown that CFA-induced inflammatory pain results in increased phosphorylation of ERK1/2 and increased expression of inflammatory mediators (TNF-α, IL-1β, and IL-6). These cytokines play major roles in the development and progression of inflammation (Calixto et al., 2004). In particular, NF-κB regulates the expression of various pro-inflammatory mediators such as cytokines, chemokines, growth factors, and inducible enzymes, which are integral to the inflammatory response (Bharti and Aggarwal, 2002). Upon NF-κB activated, it will translocates to the nucleus from the cytoplasm and activates the genes involved in inflammatory responses (Zhou et al., 2006). Therefore, inhibition of NF-κB could decrease the expression of inflammatory genes, and this may be a mechanism of action of anti-inflammatory agents (Kang et al., 2009).

Current treatments for inflammatory pain include opioids, non-steroidal anti-inflammatory drugs (NSAID), antiepileptic drugs, and other drugs, which are often associated with dose-limiting side effects (Bombardier et al., 2000; Johannes et al., 2010). Long-term use of NSAIDs may lead to adverse effects such as gastrointestinal and allergic reactions, which can limit their clinical use (Zhang et al., 2006). Various natural compounds exhibit anti-inflammatory activity through inhibition of NF-κB. For example, (Gonzales and Orlando, 2008 ) reported that curcumin and resveratrol can significantly reduce gene expression of TNF-α, IL-1β, and IL-6, resulting in decreased NF-κB activation. *Zanthoxylum nitidum (Roxb.) DC.*, also named Liang Mianzhen (LMZ), is a herb that has been used to treat injuries, rheumatism, arthralgia, stomach pain and so on in Chinese folk medicine since antiquity (Zeng et al., 1982; Hu et al., 2006; Wang et al., 2015).

Traditional Chinese medicine (TCM) is a multi-target approach that can provide significant analgesic effects on inflammatory pain with considerably fewer side effects than commercially available pain killers. The toothpaste plus LMZ is very popular in China for more than 30 years, and it has not side effect reported up to now. Based on these properties, LMZ might provide greater benefit than known analgesics for treatment of inflammatory pain. However, the mechanisms of its analgesic effect have not been reported. Since pro-inflammatory mediators such as IL-6, IL-1β, TNF-α, NF-κBp65, and p-ERK1/2 participate in descending pain inhibition, we hypothesized that LMZ may exert analgesic effects through mediating levels of NF-κBp65 and p-ERK1/2, leading to decreased levels of IL-6 and TNF-α in the spinal dorsal horn. Hence, the present investigation utilized a CFA model to evaluate the possible analgesic effects of oral LMZ administration and the associated peripheral and central mechanisms. Our work elucidated potential molecular mechanisms for the safe treatment of inflammatory pain.

## Materials and Methods

### Animals

Male C57BL/6J mice aged 8–10 weeks (Changzhou Cavens experimental animal co., Ltd., China) were housed in a temperature-controlled environment on a 12 h light/dark cycle with access to food and water. The experimental protocol followed the principles and guidelines recommended by the Chinese Society of Experimental. Animal was approved by the local Ethical Committee of the Institute. The approval number of animal ethics is 201801A002. There are 30 mice, each group contained 10 mice. All behavioral experiments were performed between 8 am and 6 pm.

### Reagents and Drugs

The extract of LMZ was purchased from Xi’an Qingzhi Bio-Tech Co., Ltd., (Xi’an, China). The standard reference substances Nitidine Chloride were purchased from National Institutes for Food and Drug Control (CAS: 110848-201604, China). CFA was purchased from Sigma–Aldrich (United States). ELISA kits for testing TNF-α (JEB-12474), IL-1β (JEB-12787), IL-6 (JEB-12267) and NF-κBp65 (JEB-15178) were obtained from Nanjing Jin Bai biological Technology Co., Ltd. The primary antibodies anti-NF-κBp65 (8242s), anti-p-ERK1/2 (4370s), anti-ERK1/2 (4695s), anti-β-actin (4970s) and the secondary antibodies HRP-conjugated anti-rabbit (7074S) were supplied by Cell Signaling Technology, United States.

### HPLC Analysis of LMZ

High Performance Liquid Chromatography (HPLC) analysis was performed using a Waters E-2695-2998 (Waters Technologies, United States), equipped with a quaternary pump (E-2695), a UV diode array detector (2998) set to collect data at 273 nm, and an autoinjector with a 2 ml sample loop. Empower software was used to control the analytical system, for data collection, and for data processing. The HPLC apparatus was equipped with a Kromasil LC-18 column (4.6 × 250 mm, 5 μm particle size) (Akzo Nobel, Sweden) kept at 25°C. The mobile phase consisted of acetonitrile (A) and 1% formic acid-triethylamine (pH = 4.5) (B), with a flow rate of 1.0 ml/min. Use the following gradient elution: 0–30 min from 20 to 50% (A) (gradient), 30–35 min 50 to 100% (A) (gradient). The sample was dissolved in methanol (0.95 mg/mL), filtered, and the obtained filtered was submitted to HPLC analysis. The peak corresponding to nitidine chloride was established by comparison of retention time and UV spectra of an authentic standard. Content was reported as percentage of nitidine chloride extracted from *Zanthoxylum nitidum (Roxb.) DC*. According to the Chinese pharmacopeia, the content of nitidine chloride should not be less than 0.13%.

### Dose Selection of LMZ

To select the most effective dose of LMZ, a 1% carrageenan-induced acute model of inflammation was induced. In accordance with a previous study (Khalid et al., 2018), we conducted a series of experiments to evaluate response to LMZ at doses of 20, 60, and 100 mg/kg against carrageenan (25 μL/paw). Mice were allocated to the following experimental groups: (A) Control, (B) Carrageenan, (C) LMZ 20 mg/kg (D) LMZ 60 mg/kg, (E) LMZ 100 mg/kg. Mice were administered intragastric vehicle (1 mL/kg, saline), or 20, 60, or 100 mg/kg LMZ, followed by a subcutaneous injection of carrageenan after 4 h. The dose that best protected against mechanical hyperalgesia and thermal hyperalgesia was chosen for further experiments.

### CFA Model of Inflammatory Pain

To establish a mouse inflammatory pain model, the mice were anesthetized with isoflurane and injected with CFA into the plantar surface of the right hind paw (Cao et al., 1998; Alhadeff et al., 2018). In the CFA and CFA+LMZ group, mice were injected with 20 μL CFA into the right hind paw and the contralateral side served as an untreated control. The control group received a saline injection into the same position. The mice were divided into the following groups: Control, CFA, and CFA+LMZ. The mice in the three groups were administered intragastric vehicle (1 mL/kg of saline) or LMZ (100 mg/kg), followed by an injection of 20 μL of CFA 1 day later.

### Behavioral Tests

#### Mechanical Hyperalgesia

We use the von Frey method to measure the mechanical hyperalgesia. In a quiet environment, the mice were placed in a special box with a mesh bottom. After 15 min of adaptation, a von Frey filament was used to stimulate the middle part of the mouse’s paw under the skin to induce a reaction. The minimal value that caused at least three responses was recorded as the mechanical pain threshold. The interval between each application was 5 min. The mechanical pain threshold was tested before the model setup, at 4 h after CFA injection, and at 4 h after daily treatment with LMZ or saline.

#### Thermal Hyperalgesia

Thermal hyperalgesia determination by hot plate apparatus, in the quiet environment, room temperature 22 ± 1°C, sets the instrument temperature to 55 ± 0.5°C. After temperature stability, mice was put in a smooth bottom of organic glass box. The reaction time for mice contraction or licking hind paws is Paw Withdrawal latency (PWL). The latency of normal mice in hot plate was 10–12 s, and the mean value of three times was taken, with each interval of 10 min. To avoid tissue damage, the upper limit of PWL was 60 s. The mice were tested, respectively, before mold making, at 4 h after CFA injection, and at 4 h after daily treatment with LMZ or saline.

#### Cold Pain Hyperalgesia

Cold hyperalgesia was measured using cold plate in the quiet environment, room temperature 22 ± 1°C, sets the instrument temperature to 4 ± 0.5°C. After the temperature was stable, the mice were placed in the bottom of a smooth plexiglass box. The times of foot lifting, licking and shaking were counted within 5 min. The mice were tested, respectively, before mold making, at 4 h after CFA injection and at 4 h after daily treatment with LMZ or saline.

### Biochemical Assays

#### Sample Collection

All experimental animals were anesthetized with isoflurane. After collecting blood samples for ELISA assays, the mice were euthanized. Tissues from the hind paw that received the injection, the L3 to L5 spinal cord (SPC), and the dorsal root ganglion (DRG) were removed and weighed, then half of the paw tissue, and all of the SPC and DRG, was snap-frozen in liquid nitrogen and stored at -80°C until further analysis. The remaining paw tissue was fixed in 10% formalin for pathology analysis.

#### Western Blot

The hindpaw tissue of the injected, L3 to L5 SPC and DRG were homogenized in RIPA Lysis Buffer. BCA Protein Assay determined protein concentrations. Equal amounts of proteins (10–40 μg) were separated by 10% SDS–PAGE and transferred onto polyvinylidene difluoride (PVDF) membranes (Millipore) at 300 mA for 70 min at 4°C. The membranes were blocked with 5% non-fat milk for 1 h at room temperature, followed by incubation with specific primary antibody overnight at 4°C. The primary antibodies used included anti-β-actin (1:1000 dilution), anti-ERK1/2 (1:1000 dilution), anti-phospho-ERK1/2 (1:1000 dilution), NF-κBp65 (1:1000 dilution). On the second day, the membranes were washed three times with TBST, and then incubated with HRP-conjugated anti-rabbit secondary antibody (1:3000 dilution) in TBST at room temperature for 1 h. The membranes were washed, and protein bands were developed using enhanced chemiluminescence (ECL) reagent (Millipore, United States), imaged with a gel imaging system (Tanon, China), and quantified using Tanon image.

#### Primer Design and Quantitative RT-PCR

Total RNA was extracted from the tissues samples using TRIzol reagent according to the manufacturer’s protocol (Takara, Japan). RNA concentrations were measured using the NanoDrop 2000 spectrophotometer (Thermo Scientific, United States). RNA (1 μg) was transcribed into cDNA by using a reverse kit (Toyobo, Japan). Then the single stranded cDNA was used as template in Real-time PCR which carried out using SYBER Green (Takara, Japan) in a Roche instrument (Roche, United States).

The primers sequence for mouse NF-κBp65, IL-6, IL-1β, TNF-α and housekeeping gene glyceraldehyde 3-phosphate dehydrogenase (G3PDH) were designed using GeneRunne. The primer sequences are as follows: The forward primer of 5′-TGA TGG TGC TGA GGG ATG CTG-3′ and reverse primer of 5′-ATT GCT GTG CCT ACC CGA AAC-3′ for NF-κBp65; The forward primer of 5′-CCC TAC TTC ACA AGT CCG GAG AGG AGA-3′ and reverse primer of 5′-GGT AGC ATC CAT CAT TTC TTT GTA TCT CT-3′ for IL-6; The forward primer of 5′-CCT GTG TCT TTC CCG TGG ACC TTC CAG G-3′ and reverse primer of 5′-CAT CAT CCC ATG AGT CAC AGA GGA TGG G-3′ for IL-1β; The forward primer of 5′-GGA ACT GGC AGA AGA GGC ACT CCC C-3′ and reverse primer of 5′-GGC CAT TTG GGA ACT TCT CAT CCC TTT G-3′ for TNF-α; The forward primer of 5′-ACC ACA GTC CAT GCC ATC AC-3′ and reverse primer of 5′-TCC ACC ACC CTG TTG CTG TA-3′for G3PDH.

The PCR program was as follows: initial denaturation at 95°C for 3 min then 95°C for 15 s and 60°C 30 s for 36 cycles (Livak and Schmittgen, 2001). SYBR was used as a fluorescent dye. Threshold cycle and Ct were measured, as they are correlate inversely with the target mRNA levels. The relative amount of gene expression, normalized to the internal control G3PDH, was calculated according to the following formula (Hussein et al., 2013):

$$\mathrm{Relative}\;\mathrm{expression}\;\mathrm{values}=2^{\left(\mathrm{Ct}\;\mathrm{G3PDH}-\mathrm{Ct}\;\mathrm{target}\;\mathrm{gene}\right)}$$

#### Enzyme-Linked Immunosorbent Assay

The paw tissue samples and serum from all groups (Control, CFA, and CFA+LMZ) were analyzed using ELISA kits. TNF-α, IL-6, IL-1β and NF-κBp65 kits were used following the manufacturer’s instructions. The paw skin tissue was quickly removed, and tissue protein extraction was performed using ice cold phosphate-buffer saline (PBS) to a final concentration of 100 mg of tissue per milliliter of PBS. The sample homogenates were then centrifuged (4°C, 3,000 rpm, 10 min). The supernatant was analyzed immediately according to the kit manufacturer’s protocols. Concentrations were determined by measuring absorbance at 450 nm. Every experiment was performed in triplicate.

#### Hematoxylin and Eosin Staining and Inflammation Scoring

Paw tissues were quickly removed and fixed in buffered 10% formalin for 24 h. Water was removed by dipping the tissues in alcohol, and then the tissues were embedded in paraffin. Sections were cut using a microtome, stained with HE, and visualized using a light microscope. The degree of inflammation was quantified using a 0 to 5 scoring system. The scores were defined as follows: 0 = no inflammation, 1 = mild inflammation, 2 = mild/moderate inflammation, 3 = moderate inflammation, 4 = moderate/severe inflammation and 5 = severe inflammation (Hussein et al., 2013).

#### Immunohistochemistry (IHC) Analysis of Tissue Sections

Paw tissues were quickly excised, fixed in buffered 10% formalin at 4°C overnight, dehydrated in a series of ethanol concentrations, cleared in xylene, and embedded in paraffin. Tissues were sectioned (4 mm), flattened, and adhered to glass slides. After dewaxing and dehydration, antigen recovery was performed by incubating the sections in pH 6.0 citric acid buffer for 10 min in 98°C water. After cooling at room temperature for 20 min, the sections were immersed in 3% hydrogen peroxide for 15 min at room temperature to abolish peroxidase activities. The sections were incubated with primary antibodies (anti-p-ERK1/2 or anti- NF-κBp65, 1:1000) in PBS for 1 h at room temperature. Then, the sections were washed with PBS and incubated for 30 min with secondary antibody at room temperature. After washing with PBS, the sections were stained with 3,3′-Diaminobenzidine peroxide (DAB) chromophore, counterstained with hematoxylin, and mounted on microscope slides for analysis. Brown staining in the cytoplasm indicated positive staining for p-ERK1/2 and NF-κBp65. The mean percentage of positively stained cells was determined by counting 1,000 stained cells in 10 different fields under 200 × magnification using a light microscope (Habib et al., 2008; Hussein et al., 2013).

#### Data Analyses and Statistics

All data were analyzed by GraphPad Prism version 7.01 and presented as mean ± SEM. *P*<0.05 means that the data is statistically significant. The data were analyzed by one-way ANOVA followed by un-paired Student’s *t*-test.

## Results

### Phytochemical Analysis of LMZ

Liang Mianzhen was analyzed by HPLC with detection at 273 nm (Supplementary Data Sheet S1). The chromatogram contained a peak corresponding to nitidine chloride at 15 min, as confirmed by comparison with calibration curves of authentic nitidine chloride standard (Supplementary Data Sheet S2). The content of nitidine chloride in the LMZ was 0.16 %, which met the specification of the Chinese pharmacopoeia. No other peaks were identified.

### Dose Selection

To select the most effective dose of LMZ, we used a carrageenan-induced model of acute inflammation. LMZ doses of 20, 60, and 100 mg/kg were selected for the study. The results showed that 20, 60 mg/kg of LMZ did not result in any significant anti-inflammatory or anti-hyperalgesic effects in the carrageenan-induced inflammatory model. However, 100 mg/kg of LMZ induced significant anti-inflammatory and anti-pain responses (Supplementary Figure S1). Therefore, 100 mg/kg LMZ was selected for further experiments.

### Effect of LMZ on CFA-Induced Mechanical Hyperalgesia

**FIGURE 1:**
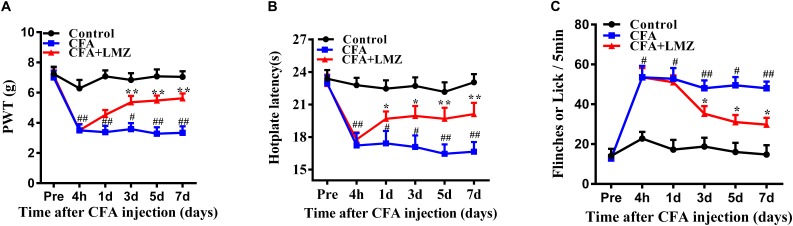
Effect of consecutive treatment LMZ or saline on CFA-induced inflammatory pain model in mice. **(A)** Injection of CFA-induced mechanical hypersensitivity in the mice paw from 1 to 7 days after CFA injection, **(B)** Thermal hyperagesia in CFA-induced, **(C)** Cold hyperagesia in CFA-induced. Data are presented as the mean ± SEM (*n* = 5). ^#^*P*<0.05, ^##^*P*<0.01, ^###^*P*<0.001 vs. Control. ^∗^*P*<0.05, ^∗∗^*P*<0.01 vs. the CFA group.

The threshold for basic mechanical paw retraction prior to implementation of the model did not differ between the groups (*P* > 0.05). The threshold for mechanical hyperalgesia significantly decreased 4 h after CFA injection (*P* < 0.01), indicating that CFA successfully induced inflammation. Compared with the CFA group, the LMZ group did not show a different threshold for mechanical hyperalgesia after the first day of administration (*P* > 0.05), but there was a significant rebound of the threshold for mechanical hyperalgesia after three days of LMZ administration (*P* < 0.05) (Figure 1A). These data indicate that LMZ prevented the development of tactile allodynia after CFA injection.

### Effect of LMZ on CFA-Induced Thermal Hyperalgesia and Cold Hyperalgesia

Liang Mianzhen significant increased hotplate latency one day after CFA injection, especially at 5 to 7 day (Figure 1B). LMZ also decreased flinches and licking frequency of mice in the LMZ group compared to that in the CFA group, especially at days five and seven (*P* < 0.05) (Figure 1C). There were no significant differences in cold hyperalgesia in response to 1 day of LMZ treatment (*P* > 0.05).

### Histological Analysis of Mouse Paw Tissues

**FIGURE 2:**
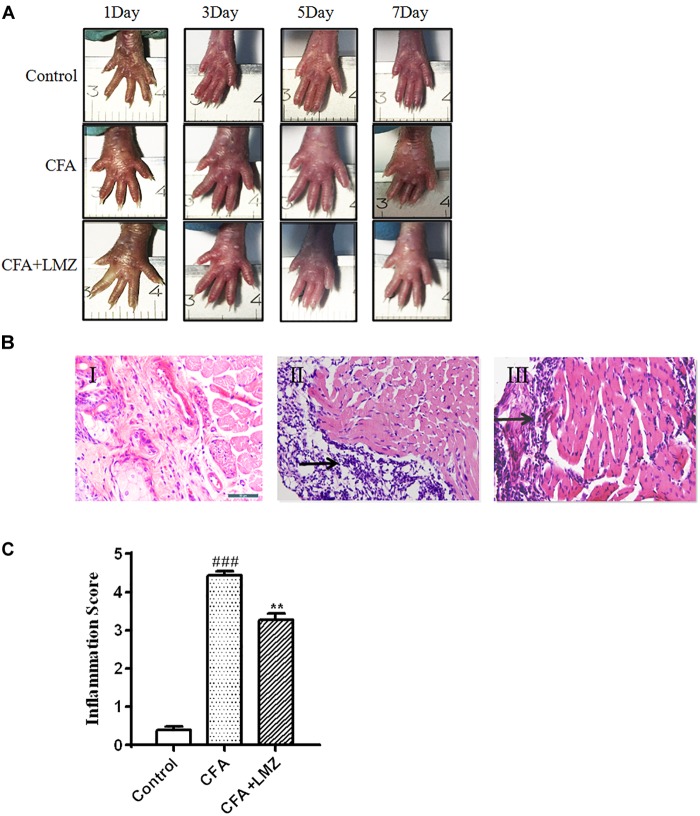
Histological evaluation of anti-inflammatory effects of LMZ in paw tissue. Hematoxylin and Eosin (HE) staining of paw tissues of mice presented as: **(A)** The representative photographs showed the plantar surface of the hinpaw after saline or CFA injection in 1, 3, 5, and 7 days. **(B)** I: Control group, II: CFA group, III: CFA+LMZ group. **(C)** Scores of inflammation in mouse paw tissues by HE staining. Each photo is representative of six specimens for each group. All figures were magnified by 400 ×. Data are presented as the mean ± SEM (*n* = 5). ^#^*P* < 0.05, ^##^*P* < 0.01, ^###^*P* < 0.001 vs. Control. ^∗^*P*<0.05, ^∗∗^*P*<0.01 vs. the CFA group.

Complete Freund adjuvant injection produced swelling on the plantar surface of the injected paw on the third day after injection. Swelling became more severe with significant inflammatory exudates at fifth day to seventh days after injection. This swelling gradually resolved within 7 days with LMZ co-administration (Figure 2A).

Histopathological examination showed that the control group fed saline had normal paw tissue (Figure 2BI). In contrast, the right hind paws of mice that received CFA injections showed massive accumulation of infiltrated cells compared to the Control group (Figure 2BII). However, inflammatory cell infiltration was significantly decreased by treatment of LMZ (100 mg/kg) on 7 day (Figure 2BIII). The degree of inflammation was evaluated by scoring of inflammation from 0 to 5. The inflammation scores indicated that co-treatment with LMZ (100 mg/kg) significantly reduced CFA-induced inflammation (Figure 2C).

### Effect of LMZ on IL-6, IL-1β, TNF-α and NF-κBp65 in CFA-Induced Mice by ELISA and qRT-PCR

To further explore the anti-inflammatory effects of LMZ, the levels of various cytokines were evaluated using ELISA and qRT-PCR.

**FIGURE 3:**
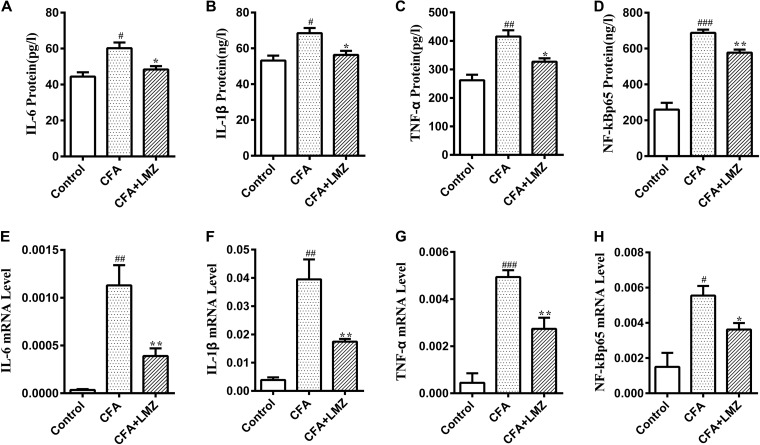
Effect of LMZ on IL-6, IL-1β, TNF-α and NF-κBp65 protein and mRNA expressions levels in mice injected paw. **(A,E)** IL-6, **(B,F)** IL-1β, **(C,G)** TNF-α and **(D,H)** NF-κBp65. Mice were treated orally LMZ (100 mg/kg) for 7 days after CFA injection. Data are presented as the mean ± SEM (*n* = 5). ^#^*P* < 0.05, ^##^*P* < 0.01, and ^###^*P* < 0.001 compare with Control; ^∗^*P* < 0.05, ^∗∗^*P* < 0.01, compare with CFA.

**FIGURE 4:**
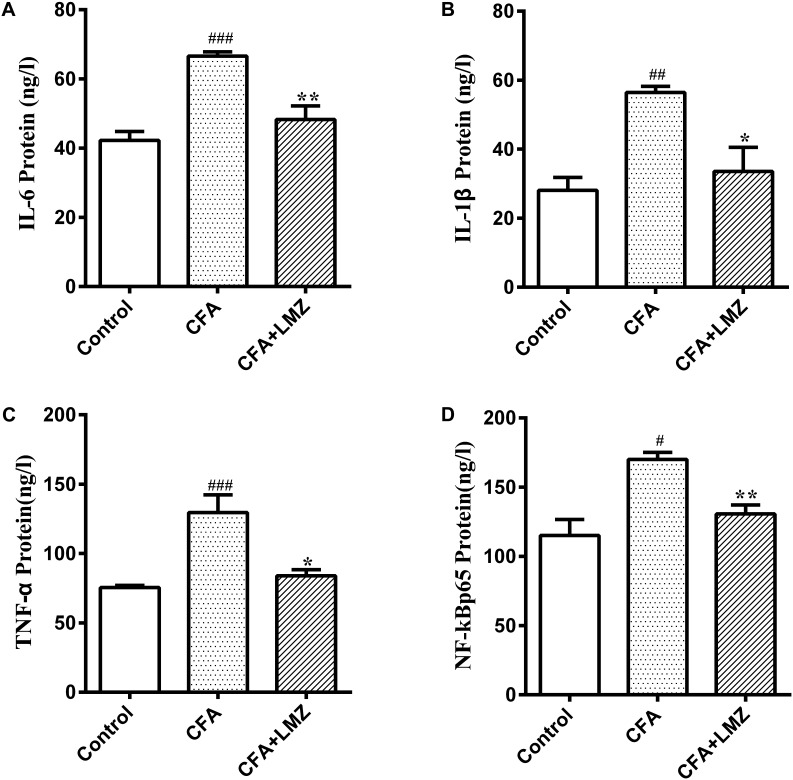
Effect of LMZ in CFA-induced mice serum. **(A)** IL-6, **(B)** IL-1β, **(C)** TNF-α and **(D)** NF-κBp65 protein concentration measured by ELISA. Mice were treated orally LMZ (100 mg/kg) for 7 days after inflammation, as induced by CFA injection. Data are presented as the mean ± SEM (*n* = 5). ^#^*P* < 0.05, ^##^*P* < 0.01, and ^###^*P* < 0.001, compare with Control; ^∗^*P* < 0.05, ^∗∗^*P* < 0.01, compare with CFA.

IL-6, IL-1β, TNF-α, and NF-κBp65 in mouse paws and serum were investigated using ELISA kits. Compared with the control group, IL-6, IL-1β, TNF-α, and NF-κBp65 protein expression was significantly increased in the CFA group 7 days after CFA injection (Figure 3A–D). However, LMZ significantly reduced expression of all of these cytokines (*P*<0.05), particularly IL-6, IL-1β, and TNF-α. Furthermore, LMZ blocked CFA-induced increases in IL-6, IL-1β, TNF-α, and NF-κBp65 in mouse serum (Figure 4).

The mRNA levels of IL-6, IL-1β, TNF-α, and NF-κBp65 were significantly increased in the paw tissue 7 days after CFA injection (Figure 3E–H). However, 7-day LMZ co-administration with CFA injection significantly reduced the expression of IL-6, IL-1β, TNF-α, and NF-κBp65 mRNA compared to the CFA group (*P*<0.05).

**FIGURE 5:**
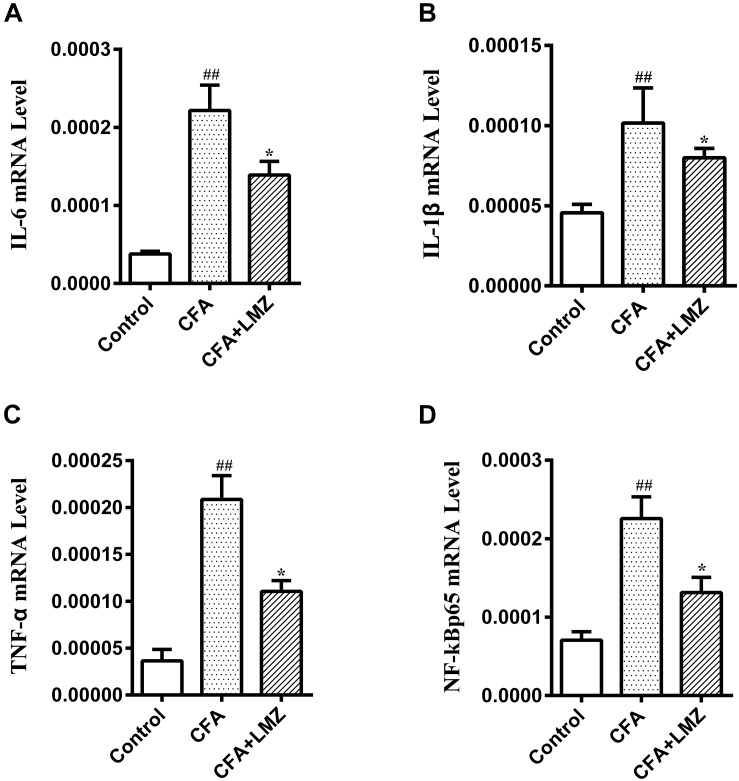
Effect of LMZ on the gene expressions in mice SPC. **(A)** IL-6, **(B)** IL-1β, **(C)** TNF-α and **(D)** NF-κBp65. Mice were treated orally LMZ (100 mg/kg) for 7 days after inflammation, as induced by CFA injection. Data are presented as the mean ± SEM (*n* = 5). ^#^*P* < 0.05, ^##^*P* < 0.01, ^###^*P* < 0.001 vs. Control. ^∗^*P* < 0.05, ^∗∗^*P* < 0.01 vs. the CFA group.

**FIGURE 6:**
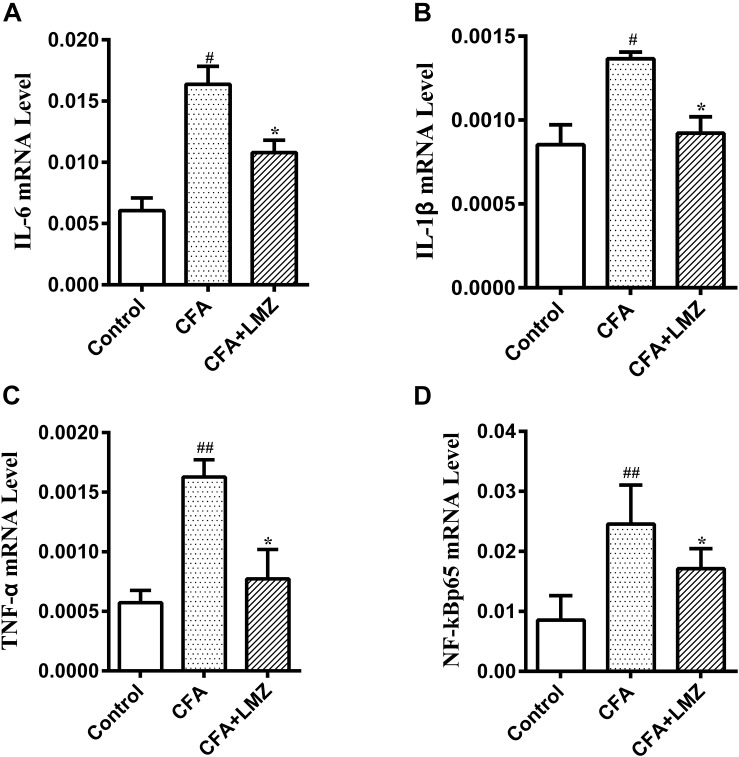
Effect of LMZ on the gene expressions in mice DRG. **(A)** IL-6, **(B)** IL-1β, **(C)** TNF-α and **(D)** NF-κBp65. Mice were treated orally LMZ (100 mg/kg) for 7 days after inflammation, as induced by CFA injection. Data are presented as the mean ± SEM (*n* = 5). ^#^*P* < 0.05, ^##^*P* < 0.01, ^###^*P* < 0.001 vs. Control. ^∗^*P*<0.05, ^∗∗^*P*<0.01 vs. the CFA group.

At the same time, the mRNA expression of IL-6, IL-1β, TNF-α, and NF-κBp65 in mice SPC and DRG were also tested by qRT-PCR. As shown in Figure 5, 6, compared with the control group, the mRNA level of IL-6, IL-1β, TNF-α, and NF-κBp65 of CFA group in mice SPC and DRG were significantly increased after 7 day of CFA injection, especially the expression of IL-1β and TNF-α. However, administration of LMZ for 7 day significantly reduced the mRNA expression level of IL-6, IL-1β, TNF-α, and NF-κBp65 in mice SPC and DRG compare to CFA group (*P*<0.05).

### The ERK1/2 and NF-κBp65 Signal Pathways May Be Involved in the Analgesic Mechanisms of LMZ

**FIGURE 7:**
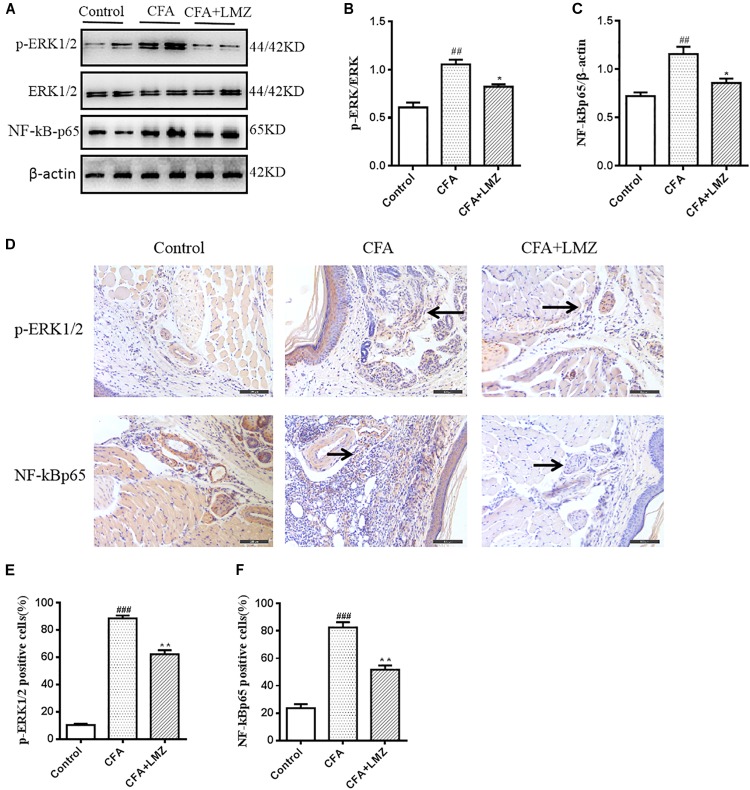
Effect of LMZ on the protein of p-ERK1/2 and NF-κBp65 expression in the paw tissues. Mice were intragastric administration with LMZ (100 mg/kg) for 7 days after inflammation as induced by CFA injection. **(A)** The representative immunoblots. **(B)** Graphic representation of relative expression of p-ERK1/2 to ERK1/2. **(C)** Graphic representation of relative expression of NF-κBp65 to β-actin. **(D)** Immunohistochemical staining for p-ERK1/2 and NF-κBp65 expression in the paw tissues. **(E)** Comparison of the percentage of cells stained with p-ERK1/2 in mice paw tissues. **(F)** Comparison of the percentage of cells stained with NF-κBp65 in mice paw tissues. The arrows indicated positive staining of p-ERK1/2 and NF-κBp65. All figures were magnified by 200 ×. Data are presented as the mean ± SEM (*n* = 5). ^#^*P* < 0.05, ^##^*P* < 0.01, ^###^*P* < 0.001 vs. control. ^∗^*P* < 0.05, ^∗∗^*P* < 0.01 vs. the CFA group.

To determine whether the analgesic effects of LMZ on mouse paw injected with CFA were associated with inhibition of the NF-κB and ERK1/2 signaling pathways, we evaluated phosphorylation of ERK and the expression of NF-κBp65 in mouse paws using western blot analysis (Figure 7A–C). The phosphorylation level of ERK1/2 and the protein expression of NF-κBp65 in the CFA group were significantly higher than those in the control group. LMZ significantly inhibited the expression of p-ERK1/2 and NF-κBp65. To further elucidate the mechanism of action in LMZ, the protein expressions of NF-κBp65 and p-EKR1/2 was determined using immunohistochemistry. As shown in Figure 7D–F, the CFA-induced inflammation group showed significantly increased expression of p-EKR1/2 or NF-κBp65 compared to the control groups. As expected, treatment with LMZ for 7 days significantly reduce p-EKR1/2 and NF-κBp65 expression when compared with the CFA group (Figure 7D). Furthermore, the percentage of cells positive for p-ERK1/2 or NF-κBp65 in mouse paw tissues was lower after administration of LMZ for 7 days (Figure 7E,F).

**FIGURE 8:**
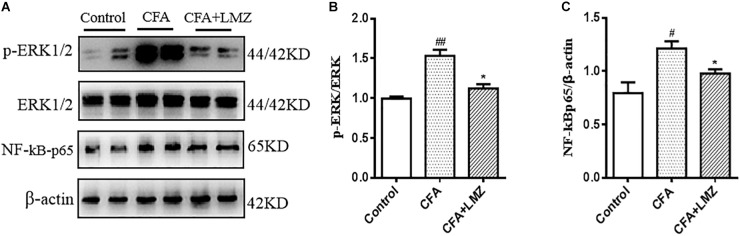
Effect of LMZ on the protein expression in mice SPC. **(A)** The representative immunoblots. **(B)** Graphic representation of relative expression of p-ERK1/2 to ERK1/2. **(C)** Graphic representation of relative expression of NF-κBp65 to β-actin. Mice were intragastric administration with LMZ (100 mg/kg) for 7 days after inflammation, as induced by CFA injection. Data are presented as the mean ± SEM (*n* = 5). ^#^*P* < 0.05, ^##^*P* < 0.01, ^###^*P* < 0.001 vs. Control. ^∗^*P*<0.05, ^∗∗^*P*<0.01 vs. the CFA group.

**FIGURE 9:**
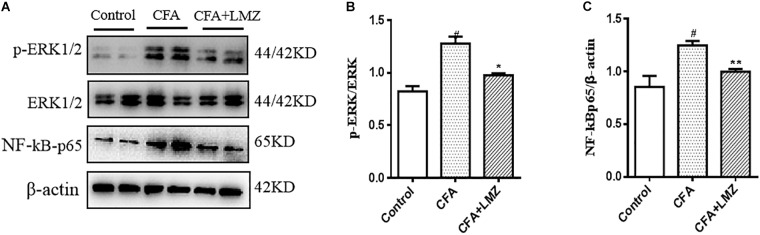
Effect of LMZ on the protein expression in mice DRG. **(A)** The representative immunoblots. **(B)** Graphic representation of relative expression of p-ERK1/2 to ERK1/2. **(C)** Graphic representation of relative expression of NF-κBp65 to β-actin. Mice were intragastric administration with LMZ (100 mg/kg) for 7 days after inflammation, as induced by CFA injection. Data are presented as the mean ± SEM (*n* = 5). ^#^*P* < 0.05, ^##^*P*< 0.01, ^###^*P* < 0.001 vs. Control. ^∗^*P* < 0.05, ^∗∗^*P* < 0.01 vs. the CFA group.

We also evaluated phosphorylation of ERK and the expression of NF-κBp65 in mouse SPC and DRG using western blot analysis (Figure 8, 9). Compared with control, the phosphorylation level of ERK1/2 and the expressions of NF-κBp65 in the CFA group were significantly up-regulated than that in the control group, especially the expression of p-ERK1/2 in mouse SPC and DRG. However, administration of LMZ for 7 day significantly down-regulate the protein expression level of p-ERK1/2 and NF-κBp65 in mice SPC and DRG compared to CFA group. Meanwhile, the relative expression of p-ERK1/2 to ERK1/2 and NF-κBp65 to β-actin were significant up-regulate in LMZ group. Thus, ERK1/2 and NF-κBp65 signal pathway involved in the analgesic effect of LMZ in peripheral and central mechanism.

## Discussion

Inflammation is often accompanied by pain. The goal of pain treatment is to reduce inflammation, which results in pain reduction. The most common treatments for inflammatory pain include NSAIDs and opioids, etc. Although these drugs are therapeutically effective, they are associated with serious side effects, such as liver and kidney damage and severe gastrointestinal reactions. Moreover, treatment of pain is often a long-term and complex process. LMZ is widely used for treatment of various arthritic diseases in TCM clinics.

Recent studies indicated that LMZ has anti-gastritis effects (Qin et al., 2016). Evidence also confirmed the gastric mucosal protection and gastrointestinal movement promotion effects of LMZ. Furthermore, a growing number of studies have revealed that LMZ has anti-cancer potency (Liu et al., 2014), gastric cancer (Shen et al., 2011), antiviral and antifungal effects (Yang and Chen, 2008), and anti-inflammatory effects (Hu et al., 2006). However, there is little research on the influences of LMZ in inflammatory pain. Thus, we studied the effects of LMZ on inflammatory pain, and explored the underlying mechanisms.

Using the CFA mice model, we found that the threshold of mechanical hyperalgesia and hotplate were markedly reduced in CFA-induced mice, and they were all increased with LMZ administration. At the same time, the cold licking frequency was increased in CFA-induced mice compared with the control mice, indicating that LMZ treatment relieves pain.

We observed that mice treated with CFA had higher level of TNF-a, IL-1β, and IL-6 in the injected paw and in the serum, while after LMZ administration, the level of TNF-a, IL-1β, and IL-6 was down-regulated. We also observed local edema of the hind paw following CFA injection, the result showed there is no significant difference between LMZ group and model group. It indicated that the peripheral inhibitory effect of LMZ on the release of inflammatory factors may not fully explain how LMZ alleviates pain. To explore the mechanisms of the effects of LMZ on inflammatory pain, we assessed central and peripheral ERK and NF-κB pathways. ERK activation has been shown to play an important role in inflammatory pain. When the receptor on the cell membrane received the inflammatory factor stimulation signal, ERK1/2 in cell cytoplasm is phosphorylated, and the signaling was transferred to the nucleus to exert its protein kinase activity. ERK is a member of MAPK family, which extensively mediates intracellular transduction of multiple signaling pathways, and has been confirmed to increase the expression of p-ERK1/2 in SPC and DRG in different pain models (Miao et al., 2015), suggesting that changes in ERK activity of SPC and DRG are closely related to the occurrence and development of neuropathic rational pain (Liu et al., 2012).

Based on the potent anti-inflammatory effects of LMZ on peripheral inflammatory conditions, and its remarkable ability to inhibit pain hyperalgesia, it is reasonable to believe that LMZ may also alleviate inflammatory pain through reduction of central inflammation. To evaluate this hypothesis, we used the hot-plate test, which evaluates activation of supraspinal structures and is a useful tool for screening analgesic drugs that producing central effects. Studies have shown that peripheral and central changes in neuropathic pain are key contributors to development of chronic pain. The SPC and DRG are believed to play key roles in development of chronic pain (Attal, 2012; von Hehn et al., 2012).

And then we performed a series of tests on the SPC and the DRG, and we found that there were varying levels of inflammatory factors and protein levels. Previous studies have shown that pro-inflammatory cytokine expression was increased in inflammatory pain (Zhu et al., 2014), as such we quantified TNF-α, IL-6, and IL-1β in the SPC and the DRG to determine whether LMZ could relieve pain by inhibiting the production of pro-inflammatory cytokines in the CNS. We found that LMZ significantly reduced the expression of TNF-α, IL-6, and IL-1β, indicating that LMZ could inhibit the expression of inflammatory cytokines in the CNS.

As established previously, the protein of IL-6, IL-1β, TNF-α and NF-κBp65 in mice serum and the mRNA level of IL-6, IL-1β, TNF-α, and NF-κBp65 in mice SPC and DRG were considerably inhibited by LMZ, it indicated LMZ’s peripheral inhibitory effect on the release of inflammatory factors may not be the entire mechanism by which it plays a role in alleviating pain. Thus, we detected the phosphorylation of ERK and the expressions of NF-κBp65 in mouse SPC and DRG by western blot analysis (Figure 8, 9). The results of this study showed that p-ERK1/2 and NF-κBp65 expression was significantly increased in the SPC and DRG of the model mice on day seven, further confirming that the activation of the SPC and DRG ERK-NF-κB was involved in the pain sensitivity regulation of the CFA model mice. At the same time, LMZ can significantly inhibit the expression of p-ERK1/2 and NF-κBp65 in the SPC and DRG. Combined with the previous behavioral test results, the authors believe that LMZ could effectively alleviate the inflammatory pain in the CFA model mice, and the mechanism may be related to the inhibition of the expression of p-ERK1/2 and NF-κBp65 in the SPC and DRG.

In summary, LMZ could inhibit activation of the ERK-NF-κB signaling pathway, and therefore may prove that LMZ is a potential therapeutic drug for the treatment of inflammatory pain. Further detailed studies aimed at isolating bioactive compounds from water-soluble LMZ extracts, and further characterization of the mechanisms of action of LMZ are needed.

## Conclusion

**FIGURE 10:**
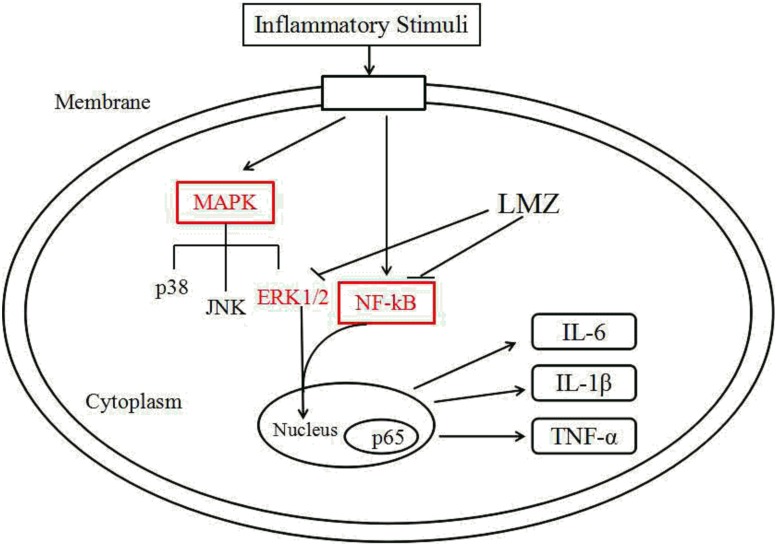
Proposed mechanisms of action by which LMZ inhibited inflammation in CFA-induced mice.

In conclusion, we reported a molecular mechanism that LMZ suppressed inflammation and ameliorated pain via central and peripheral suppression of ERK1/2 and NF-κB. After primary introduction, nociceptive stimulation can lead to central sensitization, thus stimulating the release of inflammatory cytokines in SPC. Our results demonstrated that LMZ can release inflammatory cytokines in the SPC. LMZ inhibited the production of inflammatory cytokines and inflammatory cell infiltration via suppression of the ERK1/2 and NF-κB signaling pathways (Figure 10). Our results suggested that LMZ is a potential candidate drug for management of chronic inflammatory pain in the clinically.

## Ethics Statement

This study was carried out in accordance with the recommendations of the “Chinese Society of Experimental Animals.” The protocol was approved by the Committee on the Ethics of Animal Experiment of Nanjing University of Chinese Medicine, Nanjing, China. All animal was approved by the local Ethical Committee of the Institute. The approval number of animal Ethics is 201801A002.

## Author Contributions

FQ participated in designing experiments, carried out the experiments in this study, prepared the first draft and revising of this manuscript. ZL and XL conceived of the study, participated in its design. HZ, AL, and QS performed a part of experiments. QW and SL participated in experimental design and consulted on the study. QL and HG offered the experiments technique on the study. All authors approved the final manuscript.

## Conflict of Interest Statement

The authors declare that the research was conducted in the absence of any commercial or financial relationships that could be construed as a potential conflict of interest.

## Supplementary Material

DATA SHEET S1 HPLC chromatogram of the methanolic crude extract of LMZ. The nitidine chloride is indicated with an arrow.

DATA SHEET S2 HPLC chromatogram of nitidine chloride.

FIGURE S1 The dose response of LMZ at the doses of 20, 60, 100 mg/kg against carrageenan-induced inflammatory pain model in mice. For dose response, mice were allocated to experimental groups: Control, carrageenan, carrageenan+LMZ 20 mg/kg, carrageenan+LMZ 60 mg/kg, carrageenan+LMZ 100 mg/kg. After 4 h of carrageenan administration, **(A)** mechanical hypersensitivity, **(B)** thermal hyperagesia were measured. Data are presented as the mean ± SEM (*n* = 5). ^∗^*P* < 0.05, ^∗∗^*P* < 0.01 vs. the carrageenan group.

## Abbreviations

CFA: complete Freund’s adjuvant
CREB: cAMP-Response Element Binding protein
DRG: Dorsal root ganglia
ELISA: Enzyme Linked Immunosorbent Assay
ERK: Extracellular regulated protein kinases
HE: Hematoxylin and eosin
IL-1β: Interleukin-1β
IL-6: Interleukin- 6
LMZ: *Zanthoxylum nitidum DC (Rutaceous)*, named Liang Mianzhen
MAPK: Mitogen-Activated Protein Kinase
NF-κBp65: Nuclear factor-κB
NSAID: non-steroidal anti-inflammatory drug
PBS: Phosphate Buffer Saline
PWL: Paw Withdrawal Latency
qPCR: Real-time Quantitative PCR
SPC: Spinal cord
TNF- α: Tumor necrosis factor-α
