# Supplementary figures and images for: Analgesic Effect of Zanthoxylum nitidum Extract in Inflammatory Pain Models Through Targeting of ERK and NF-κB Signaling
Source: Front Pharmacol. 2019 Apr 24;10:359. doi: 10.3389/fphar.2019.00359 (PMC6491746; doi:10.3389/fphar.2019.00359)

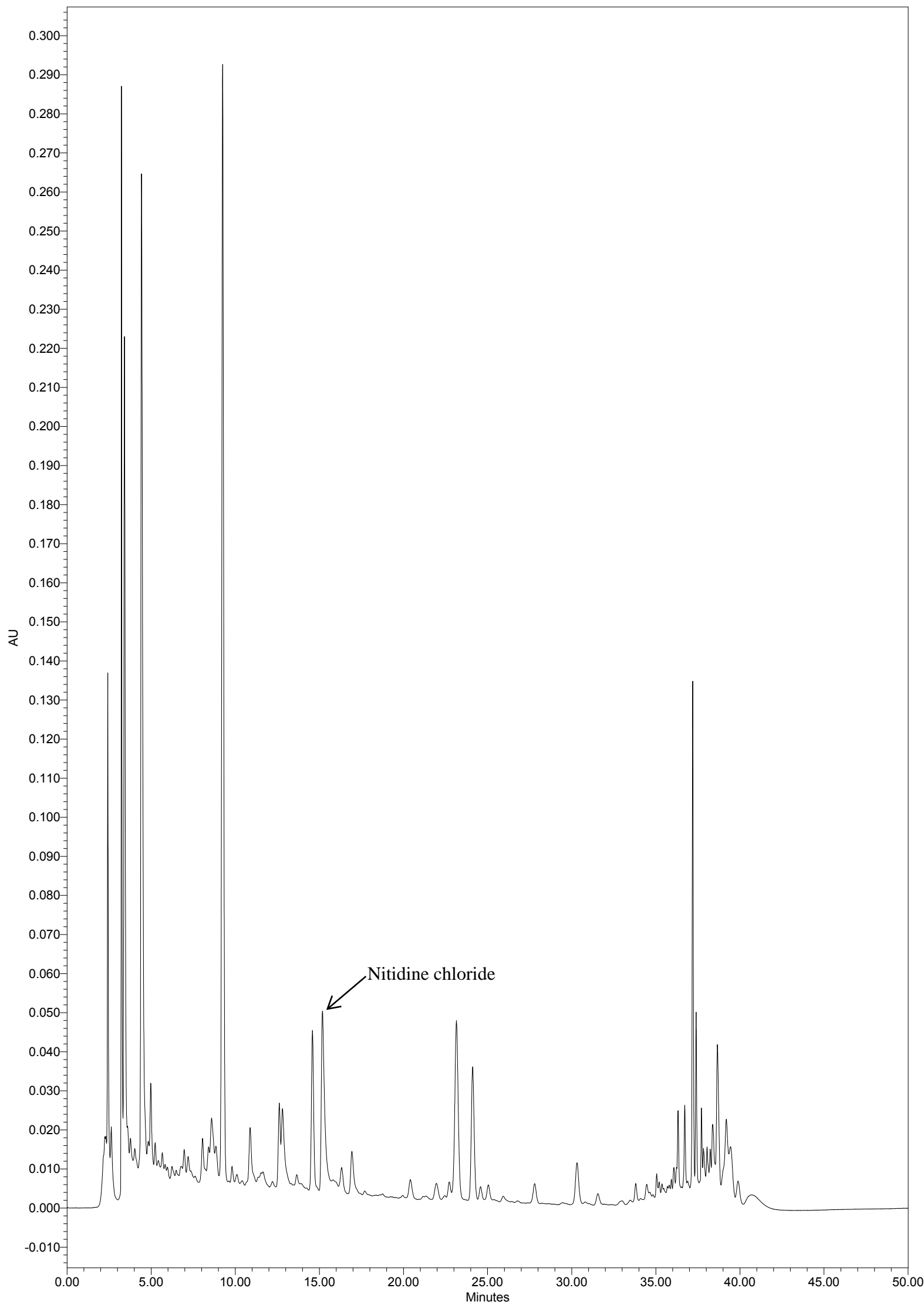

Supplement: DATA SHEET S1 — HPLC chromatogram of the methanolic crude extract of LMZ. The nitidine chloride is indicated with an arrow. [file Data_Sheet_1.PDF]

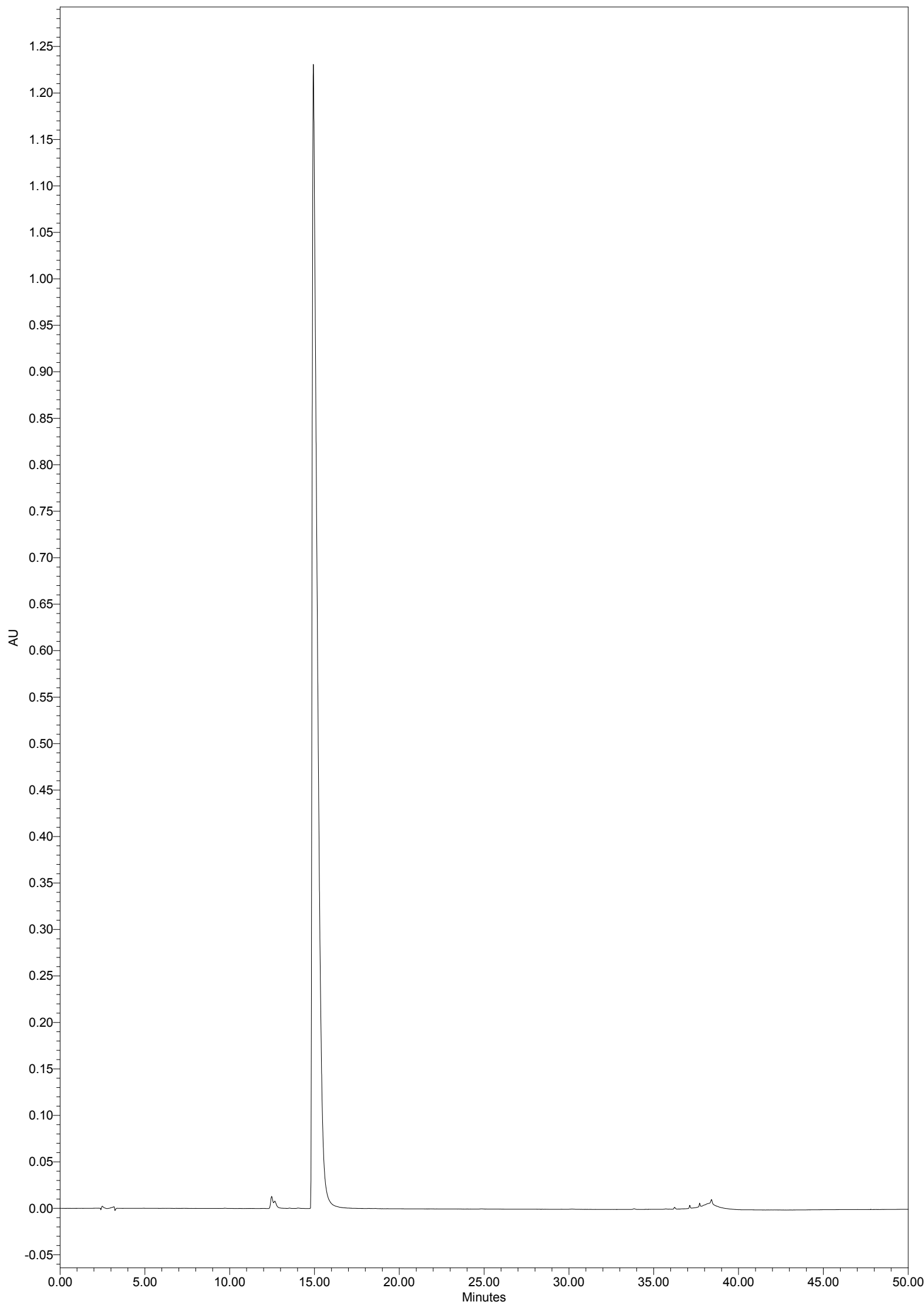

Supplement: DATA SHEET S2 — HPLC chromatogram of nitidine chloride. [file Data_Sheet_2.PDF]
